# Pressure Analysis of Dynamic Injection Molding and Process Parameter Optimization for Reducing Warpage of Injection Molded Products

**DOI:** 10.3390/polym9030085

**Published:** 2017-03-07

**Authors:** Xinyu Wang, Hongxia Li, Junfeng Gu, Zheng Li, Shilun Ruan, Changyu Shen, Minjie Wang

**Affiliations:** 1 School of Mechanical Engineering, Dalian University of Technology, Dalian 116023, China; seringwang@dlut.edu.cn (X.W.); hxli@dlut.edu.cn (H.L.); mjwang@dlut.edu.cn (M.W.); 2 Department of Engineering Mechanics, State Key Laboratory of Structural Analysis for Industrial Equipment, Dalian University of Technology, Dalian 116023, China; jfgu@dlut.edu.cn (J.G.); ruansl@dlut.edu.cn (S.R.); shency@dlut.edu.cn (C.S.); 3 National Center for International Research of Micro-nano Molding Technology & Key Laboratory for Micro Molding Technology of Henan Province, Zhengzhou 450001, China

**Keywords:** dynamic injection molding, warpage optimization, pressure response analysis, kriging model, EGO method

## Abstract

Plastic injection molding technology is one of the important technologies for the manufacturing industry. In this paper, a numerical dynamic injection molding technology (DIMT) is presented, which is based on the finite element (FE) method. This numerical simulation method introduces a vibrational force into the conventional injection molding technology (CIMT). Some meaningful work has been executed for investigating the mechanical evolution behavior of DIMT. As the basis for illustrating the mechanism in warpage optimization results, dynamic parameter analysis on the rule of pressure response is performed in detail. In the warpage optimization work, three kinds of structure with different molding materials are selected as the comparison. The final warpage of each product is efficiently minimized by using a Gaussian process-based sequential optimization method. From the further discussions, the features of DIMT in improving the molding quality are revealed, indicating that it may not be appropriate for geometrically large structures. This study has significant meaning for the actual injection molding industry.

## 1. Introduction

Plastic injection molding technology has been regarded as one of the most important technologies for rapid, efficient manufacturing. During the injection molding process, a plastic melt is injected into the void cavity by the screw of the injection molding machine, and subsequently solidified in the cavity after a certain cooling time. It is a complicated multi-physical process involving a thermal and mechanical evolution history. The level of molding defects, like warpage or shrinkage magnitude, length of weld line, etc., are associated with this complicated thermal and mechanical history. Therefore, it has attracted researchers’ attention to investigate the thermal and mechanical behavior in the injection molding process, and relevant process optimization methods to improve the molding quality for a long time.

In fact, the thermal and mechanical behavior of the plastic material depends on the process parameters, molding temperature, pressure, etc. As a special injection molding technology, dynamic injection molding technology (DIMT) integrates melt vibration technology [1] and injection molding technology. It attempts to introduce the vibrational force field into plasticizing, filling, and packing stages to disturb the flow behavior [2,3], crystallization behavior, etc. The final internal stress field may be more proper to eliminate the defects of the product. The mechanism is that the thermal and mechanical evolution history is changed due to this external vibration.

Many studies have introduced the research work of the DIMT effect on the mechanical or electronic properties of plastics from the experimental perspective. For instance, Kikuchi et al. [4] improved the mechanical strength of plastics by using DIMT, and observed the alternation of residual stress distribution of the injected samples from the birefringence pattern. Yan et al. [5] observed the shear stress and apparent viscosity reduction with DIMT. Yang et al. [6] undertook detailed research on the electrical conductivity of the carbon nanotube/polypropylene composite with the help of DIMT, and observed the perfect conductivity paths under DIMT which can improve the electrical conductivity. Additionally, Yin et al. [7] investigated the pressure response and crystallization behavior, and found that the crystallization is increased under DIMT. Wang et al. [8] improved the properties of CaCO_3_/PP composite material by using DIMT in plasticizing. However, there is a lack of literature on DIMT from a simulation point of view to investigate the thermal and mechanical evolution history, revealing a mechanism for improving the molding quality of plastic products.

Perfect molding quality cannot be obtained without the proper molding process parameters. For thin-walled products, warpage is one main molding defects. The main work of many studies is to find the optimum manufacturing process conditions to minimize the warpage. The method is to find a bridge to combine the computer aided engineering (CAE) technology with the mathematical optimization theory. For instance, Ozcelik and Erzurumlu [9] used artificial neural network (ANN)- and genetic algorithm (GA)-based optimization methods to minimize the warpage of the product. Kitayama et al. [10] reduced the warpage of the product by optimizing the packing pressure curve. Zhou and Turng [11] proposed a process parameters optimization method on warpage and volumetric shrinkage with the help of a Gaussian process surrogate model. Wu, Ku, and Pai [12] reduced the warpage of the injection molding product with weld line constraints. Gao and Wang [13,14] employed an efficient adaptive optimization method to reduce the warpage defect, which is based on a kriging surrogate model and expected improvement (EI) function. Shi et al. [15,16] used an ANN model to optimize the warpage of the product. Farshi et al. [17] minimized both the warpage and the volumetric shrinkage by using the sequential variable-size simplex algorithm. Moreover, considering multi-class design variables, including process parameters, geometry information of the gate location, and thickness of the part, Deng et al. [18] employed a particle swarm optimization (PSO) algorithm for solving the multi-objective problem including warpage, weld line, and air traps. Zhao et al. [19] solved the multi-objective optimization problem of an injection molding product, including warpage, shrinkage and sink marks. However, this literature is all on the basis of the conventional injection molding technology (CIMT).

In our previous work [20], we proposed a numerical method to simulate the process of DIMT and performed the process parameter optimization to minimize the warpage of a typical product. This paper is aimed at trying to extend our previous work to make an in-depth and detailed study on the mechanical evolution behavior and reveal the advantage and disadvantage for improving the molding quality. The implementation of numerical DIMT by using the finite element method is introduced with the given assumptions and solution conditions in the subsequent section. Then, pressure response analysis is performed in order to discuss the influence of dynamic parameters on the pressure field. This can help us to realize the mechanical evolution rules and clarify the mechanism for improving the molding quality. The most significant thing is to further argue the actual effect on warpage reduction by using a kriging model-based sequential optimization method. It is meaningful for engineers to recognize the features of DIMT when they produce products with DIMT. These are the differences and innovative aspects of this paper compared with our previous study of the literature [20].

## 2. Flow Analysis Computation Method of Dynamic Injection Molding Technology (DIMT)

### 2.1. Fundamental Physical Equations

During the whole injection molding processing, the flow process is the early stage, which can determine the molding quality in the late stage. It can be divided into the filling phase and packing phase. In the filling phase, the plastic melt is injected into the void mold cavity. If the mold cavity is almost filled with the plastic material, the packing process proceeds. During the packing phase, there is still a certain amount of plastic material being injected into the cavity, which ensures filling the gap due to material shrinkage from the melt state to solid state. Although the molding process of the product is a complicated physical process, it should also satisfy the fundamental physical governing equations.

Equations (1)–(3) are the complete governing equations to describe the physical change of the plastic melt during injection molding, including the change of mass, momentum and energy.

(1) $$\frac{1}{\rho }(\frac{\partial \rho }{\partial t}+v\cdot \nabla \rho )+\nabla \cdot v=0$$

(2) $$\rho (\frac{\partial v}{\partial t}+v\cdot \nabla v)=\nabla \cdot \sigma +\rho \cdot f$$

(3) $$\rho c_{p}(\frac{\partial T}{\partial t}+v\cdot \nabla T)=\beta T(\frac{\partial p_{e}}{\partial t}+v\cdot \nabla p_{e})+\tau :\nabla v\;+\kappa \cdot {\left(\nabla \cdot v\right)}^{2}+\nabla \cdot (k\nabla T)\;+\rho \cdot \dot{q}$$

(4) σ=−pI+τ

where *ρ*, *c*_p_, *β*, and *k* represent four physical properties, density, specific heat, coefficient of thermal expansion, and thermal conductivity, respectively. ***v*** and ***f*** represent the velocity field and body force per unit mass, respectively. *σ* is the stress tensor that can be decomposed into an isotropic part and a non-isotropic part, as in Equation (4). $\dot{q}$ is the heat generation rate per unit mass. *κ* is the expansion viscosity that can determine the heat generation level during expansion. *T* and *p_e_* represent the temperature and equilibrium pressure, respectively.

Obviously, it is difficult to solve the complete equations above, so we have to make some physical assumptions for simulating the plastic injection molding process. The main assumptions are:

(a) For thin-walled injection molded products, the flow behavior of the melt can be regarded as the general Hele-Shaw flow, which neglects the “fountain flow” phenomenon at the front of the melt. The finite mesh type is the mid-plane mesh with triangular elements.
(b) Considering the high viscosity effect of the plastic melt, inertia force and gravity are neglected. Therefore, the flow process of the melt is treated as a laminar flow, and regarded as the general Newtonian fluid. The melt is dominated by shear stress.
(c) No-slip assumption. This means that there is no relevant movement between the melt and the mold cavity surface, or between the melt and the solid plastic material surface.
(d) For the energy transport issue, there is no heat source in the melt in practice. We only consider heat conduction, but neglect heat convection along the thickness direction. In contrast, we consider heat convection and neglect heat conduction along the flow direction.

According to Assumption (b), the constitutive equation of the melt should coincide with the form of the Newtonian fluid, which reads as:

(5) $$\tau_{ij}=\eta \left(T,p,\dot{\gamma }\right)\cdot {\dot{\gamma }}_{ij}$$

where ${\dot{\gamma }}_{ij}$ is the rate of strain tensor. *η*(*T*, *p*, $\dot{\gamma }$) is the viscosity of the melt, and is a highly non-linear function on temperature *T*, pressure *p*, and shear rate $\dot{\gamma }$, which is defined as:

(6) $$\dot{\gamma }=\sqrt{\frac{1}{2}\sum_{i=1}^{3}\sum_{j=1}^{3}{\dot{\gamma }}_{ij}{\dot{\gamma }}_{ji}}$$

Many researchers made efforts in order to obtain a perfect model of *η*(*T*, *p*, $\dot{\gamma }$) [21,22,23], listed in Table 1. However, the Cross-WLF model is suitable to be used as a computation model for most plastic materials. In this paper, Cross-WLF model is employed.

By using the assumptions above, the momentum equation and energy equation are simplified as:

(7) $$\{\begin{array}{c} \frac{\partial p}{\partial x}=\frac{\partial }{\partial z}\left(\eta \cdot \frac{\partial v_{x}}{\partial z}\right)\\ \frac{\partial p}{\partial y}=\frac{\partial }{\partial z}\left(\eta \cdot \frac{\partial v_{y}}{\partial z}\right) \end{array}$$

(8) $$\{\begin{array}{ll} \rho_{m}c_{pm}\left(T\right)\left(\frac{\partial T}{\partial t}+v_{x}\frac{\partial T}{\partial x}+v_{y}\frac{\partial T}{\partial y}\right)=\eta \cdot {\dot{\gamma }}^{2}+\frac{\partial }{\partial z}\left(k_{m}\left(T\right)\frac{\partial T}{\partial z}\right) & \mathrm{For}\;\mathrm{melt}\;\mathrm{state}\\ \rho_{s}c_{ps}\left(T\right)\frac{\partial T}{\partial t}=\frac{\partial }{\partial z}\left(k_{s}\left(T\right)\frac{\partial T}{\partial z}\right) & For\;solid\;state \end{array}$$

In order to solve these governing equations, it should combine with the solution conditions include the conditions of pressure, velocity, continuity and temperature:

(9) $$\{\begin{array}{ll} p=p_{inlet}\left(t\right) & At\;the\;gate\\ p=0 & At\;the\;front\;of\;the\;melt\\ \frac{\partial p}{\partial n}=0 & No\;\mathrm{penetration}\;conditon \end{array}$$

(10) $$\{\begin{array}{ll} v_{z}=0 & \\ v_{x}=v_{y}=0 & when\;\xi \leq \left|z\right|\leq h\\ \frac{\partial v_{x}}{\partial z}=\frac{\partial v_{y}}{\partial z}=0 & when\;z=0 \end{array}$$

(11) $$\{\begin{array}{ll} \dot{m}={\dot{m}}_{inlet}\left(t\right) & If\;given\;flow\;rate\;condition\;at\;the\;gate\\ \dot{m}=0 & Net\;flow\;rate\;in\;the\;melt \end{array}$$

(12) $$\{\begin{array}{ll} T=T_{wall} & Wall\\ T=T_{\mathrm{melt}} & Gate\\ T=T_{upstrom\;core} & Front\;of\;the\;melt\\ \frac{\partial T}{\partial z}=0 & at\;the\;node\;of\;the\;mid-plane\;mesh \end{array}$$

By means of integrating the continuous Equation (1) along with the thickness and Galerkin-weighted residual method, we can obtain Equation (13) completely for solving the flow process:

(13) $$\int_{\Omega }w\cdot \left[G\frac{\partial p}{\partial t}+\nabla \cdot \left(S\cdot \nabla p\right)+F\right]d\Omega +\int_{\Gamma }\bar{w}\cdot \left[\left(S\cdot \nabla p\right)\cdot n-\bar{m}\right]d\Gamma =0$$

where:

$$G={\int_{0}^{\xi }\frac{\partial \rho_{m}}{\partial p}|}_{T}dz+{\int_{\xi }^{h}\frac{\partial \rho_{s}}{\partial p}|}_{T}dz\;S=\int_{0}^{\xi }\rho \int_{-\xi }^{\tilde{z}}\frac{z\prime }{\eta }dz\prime d\tilde{z}\;F={\int_{0}^{\xi }\frac{\partial \rho_{m}}{\partial T}|}_{p}\frac{\partial T}{\partial t}dz+{\int_{\xi }^{h}\frac{\partial \rho_{s}}{\partial T}|}_{p}\frac{\partial T}{\partial t}dz+{\left(\rho_{m}-\rho_{s}\right)}_{z=\xi }\frac{\partial \xi }{\partial t}$$

where *ξ* is the position between the melt and solid material along the thickness.

In addition, many properties of the plastic material are variable with temperature and pressure, like thermal conductivity and density. For describing the thermal conductivity evolution, we employ:

(14) $$k\left(T\right)=\lambda_{1}+\lambda_{2}\left(T-\lambda_{5}\right)+\lambda_{3}\tanh\left[\lambda_{4}\left(T-\lambda_{5}\right)\right]$$

where *λ*_1_ to *λ*_5_ are constant parameters.

**Figure 1:**
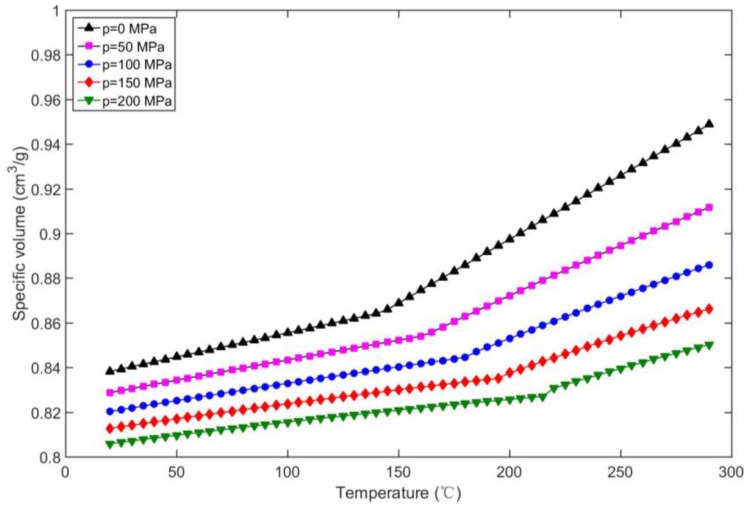
Specific volume vs. temperature of PC Lexan 105.

The pressure-volume-temperature relationship, which is usually called the pvT state equation, is the critical rule to characterize the change of density (specific volume) with respect to temperature and pressure. For the pvT state equation, there are several state models to describe the property change, like the Spencer-Gilmore (S-G) model [24] and the two-domain Tait model [25]. In this study, we employ the two-domain Tait model, as Equation (15) expresses:

(15) $$\upsilon \left(T,\;p\right)=\upsilon_{0}\left(T\right)\left[1-C\ln\left(1+\frac{p}{B\left(T\right)}\right)\right]+\upsilon_{t}\left(T,\;p\right)$$

where *C* = 0.0894, and:

(16) $$\upsilon_{0}\left(T\right)=\{\begin{array}{ll} b_{1m}+b_{2m}\left(T-b_{5}\right) & if\;T>T_{t}\\ b_{1s}+b_{2s}\left(T-b_{5}\right) & if\;T<T_{t} \end{array}$$

(17) $$B\left(T\right)=\{\begin{array}{cc} b_{3m}\exp\left[-b_{4m}\left(T-b_{5}\right)\right] & if\;T>T_{t}\\ b_{3s}\exp\left[-b_{4s}\left(T-b_{5}\right)\right] & if\;T<T_{t} \end{array}$$

(18) $$\upsilon_{t}\left(T,\;p\right)=\{\begin{array}{ll} 0 & if\;T>T_{t}\\ b_{7}\exp\left[b_{8}\left(T-b_{5}\right)-b_{9}p\right] & if\;T<T_{t} \end{array}$$

(19) $$T_{t}\left(p\right)=b_{5}+b_{6}\cdot p$$

where *b*_1_ to *b*_9_ are material parameters, *T_t_* represents the transition temperature, which is a crystalline temperature for crystalline plastic and the glass transition temperature for amorphous plastic. As an explanation of the pvT relationship, Figure 1 presents the specific volume change of PC Lexan 105 with respect to temperature and pressure.

### 2.2. The Finite Element Equation for Computing the Pressure Field

For simulating the flow process during injecting, we need to deduce the finite element discrete form of Equation (13). Consider that the pressure in any element is interpolated by:

(20) $$p\left(x,y,t\right)=\sum_{m=1}^{3}L_{m}\left(x,y\right)p_{m}\left(t\right)$$

where index *m* indicates the node number of the current element. *L_m_* is the interpolation function, which satisfies:

(21) $$\left[\begin{array}{c} L_{i}\\ L_{j}\\ L_{k} \end{array}\right]=\frac{1}{2A}\left|\begin{array}{ccc} x_{j}y_{k}-x_{k}y_{j} & y_{j}-y_{k} & x_{k}-x_{j}\\ x_{k}y_{i}-x_{i}y_{k} & y_{k}-y_{i} & x_{i}-x_{k}\\ x_{i}y_{j}-x_{j}y_{i} & y_{i}-y_{j} & x_{j}-x_{i} \end{array}\right|\left[\begin{array}{c} 1\\ x\\ y \end{array}\right]=\frac{1}{2A}\left|\begin{array}{ccc} a_{i} & b_{i} & c_{i}\\ a_{j} & b_{j} & c_{j}\\ a_{k} & b_{k} & c_{k} \end{array}\right|\left[\begin{array}{c} 1\\ x\\ y \end{array}\right]$$

where *A* is the area of the triangle element. Then, we can obtain the finial Equation (22):

(22) $$\left[\begin{array}{ccc} K_{GG} & K_{GM} & K_{GF}\\ K_{MG} & K_{MM} & K_{MF}\\ K_{FG} & K_{FM} & K_{FF} \end{array}\right]\left[\begin{array}{c} P_{G}\\ P_{M}\\ P_{F} \end{array}\right]=\left[\begin{array}{c} Q_{G}\\ Q_{M}\\ Q_{F} \end{array}\right]$$

where ***K***, ***P***, and ***Q*** represent the stiffness matrix, pressure vector, and the net flow rate vector, respectively. Subscript *G*, *M*, and *F* represent the injecting gate, melt region and the flow front of the melt, respectively. Therefore, ***Q****_M_* = 0 in the melt, and ***P****_F_* = 0 at the front of the melt.

For the conventional injection molding, flow rate ***Q****_G_* is usually a given constant during filling analysis, and ***P****_G_* is a given constant or linear form during the packing phase. We can make a slight change of Equation (22):

(23) $$\{\begin{array}{l} K_{GG}P_{G}+K_{GM}P_{M}=Q_{G}^{Const}+{\tilde{Q}}_{G}\left(t\right)\\ K_{MG}P_{G}+K_{MM}P_{M}=0\\ K_{FG}P_{G}+K_{FM}P_{M}=Q_{F} \end{array}$$

for computing the pressure and velocity field in filling analysis with ***Q****_G_* given, and:

(24) $$\{\begin{array}{l} K_{GG}\left(P\prime \left(t\right)+\tilde{P}\left(t\right)\right)+K_{GM}P_{M}=Q_{G}\\ K_{MG}\left(P\prime \left(t\right)+\tilde{P}\left(t\right)\right)+K_{MM}P_{M}=0\\ K_{FG}\left(P\prime \left(t\right)+\tilde{P}\left(t\right)\right)+K_{FM}P_{M}=Q_{F} \end{array}$$

for computing the pressure and velocity field in packing analysis with ***P****_G_* given.

For the dynamic filling phase, the flow rate can be deduced by the advancing displacement of the screw which is:

(25) $$S\left(t\right)=v_{ave}\cdot t+S_{amp}\cdot \sin\left(2\pi f_{fill}\cdot t+\varphi_{fill}\right)$$

where *v_ave_* is the average advancing velocity of the screw, *S_amp_*, *f_fill_*, and *φ_fill_* are amplitude, frequency, and phase of a vibration filling. Then, the flow rate is:

(26) $$\begin{array}{cc} Q\left(t\right) & =A_{sc}v_{ave}\left[1+\varsigma \cdot \cos\left(2\pi f_{fill}\cdot t+\varphi_{fill}\right)\right]\\ & =\frac{V}{t_{fill}}\cdot \left[1+\varsigma \cdot \cos\left(2\pi f_{fill}\cdot t+\varphi_{fill}\right)\right]\\ & =Q_{const}+\tilde{Q}\left(t\right) \end{array}$$

where *V* is the volume of the product, *A*_sc_ here is the area of the screw, *t_fill_* is the approximate filling time. *ς* equals (*S_amp_*∙2π∙*f_fill_*∙*t_fill_*∙*A_sc_*)/*V* herein.

Although Equation (26) is available to be the dynamic filling process, there is a limitation that, in order to guarantee no melt reflux problems, (*S_amp_*∙2π∙*f_fill_*∙*t_fill_*∙*A_sc_*)/*V* ≤ 1 has to be satisfied. This indicates that high frequency or high amplitude vibration cannot be realized, since if *S_amp_* or *f_fill_* is higher, *f_fill_* or *S_amp_* will be so low that the vibration will be “no sense” in practice. Therefore, we prefer to choose another case of dynamic filling rather than Equation (26) in the following study.

Therefore, if we express the advancing velocity of the screw in a simple form as:

(27) $$v\left(t\right)=v_{ave}\left[1+\varepsilon \cdot \sin\left(2\pi f_{fill}\cdot t+\varphi_{fill}\right)\right]$$

where *ε* is called the amplitude factor, we can easily obtain the dynamic filling flow rate expression as:

(28) $$\begin{array}{cc} Q\left(t\right) & =\frac{V}{t_{fill}}\cdot \left[1+\varepsilon \cdot \sin\left(2\pi f_{fill}\cdot t+\varphi_{fill}\right)\right]\\ & =Q_{const}+\tilde{Q}\left(t\right) \end{array}$$

Equation (28) avoids the constraint problem mentioned above, and can be realized easily as well. For the packing phase, making the similar manipulation the filling phase, the packing pressure curve is controlled by:

(29) $$P_{packing}=P_{final}\cdot \left[\left(P_{start}-\delta \cdot t)+\theta \cdot \sin(2\pi f_{pack}\cdot t+\varphi_{pack}\right)\right]$$

where *P_final_* represents the finial pressure during the filling phase, *P_start_* is the packing pressure fraction at the beginning, *δ* is a factor to control the trend of the packing curve, *θ*, *f_pack_*, and *φ_pack_* represent the amplitude, frequency, and phase of a vibration term, respectively.

Overall, with Equations (28) and (29), Equations (23) and (24) can be solved to investigate the pressure response analysis and the molding quality improvement analysis of plastic product of numerical DIMT, which will be argued in the following sections.

## 3. Pressure Response Analysis in the Cavity

Defects, like warpage, shrinkage, etc., depend on the mechanical and thermal evolution history. By integrating Equation (7) associated with the solution conditions, we can deduce the velocity formula as:

(30) $$v_{x}=\{\begin{array}{cc} \frac{\partial p}{\partial x}\int_{-\xi }^{\tilde{z}}\frac{z\prime }{\eta }dz\prime & \left|z\right|<\xi \\ 0 & \xi \leq \left|z\right|\leq h \end{array}$$

(31) $$v_{y}=\{\begin{array}{cc} \frac{\partial p}{\partial y}\int_{-\xi }^{\tilde{z}}\frac{z\prime }{\eta }dz\prime & \left|z\right|<\xi \\ 0 & \xi \leq \left|z\right|\leq h \end{array}$$

where *h* is the thickness of the product.

Obviously, solving the velocity field requires us to firstly solve the pressure field. Moreover, computing the heat contribution from shear motion relies on the pressure field as well. Therefore, pressure in the melt is a very important mechanical quantity to lead the material motion and the thermal evolution. This indicates that pressure response analysis can help us to understand the mechanical and thermal evolving history.

**Figure 2:**
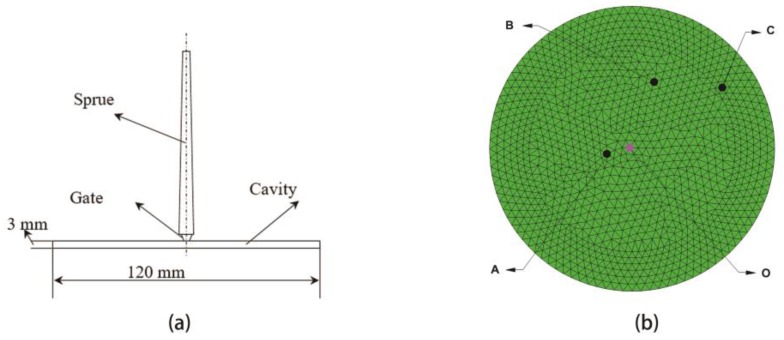
The geometry structure of the low-density polyethylene (LDPE) disk (**a**) and the finite element model (**b**): O is the location of the injecting gate, A, B and C are three monitor points, and OA, OB, and OC are 10.08, 29.40 and 46.34 mm, respectively.

Therefore, a finite element model of a low-density polyethylene (LDPE) circle disk product (depicted as Figure 2) is modeled as the investigating objective, which is the same as the product of Yin’s experiment [7]. The basic material properties of LDPE are listed as Table 2. The injecting gate is located at the center of the disk, which is colored with pink in the finite element model.

In order to compare the simulation result of DIMT with the experimental observation [7], three monitor points, except the gate, A, B, and C, are selected in Figure 2b. The distance of OA, OB, and OC are 10.08, 29.40, and 46.34 mm, respectively. The melt temperature and mold temperature are 190 °C and 50 °C, respectively. Moreover, injecting time and packing time are both 2 s. Other dynamic parameters are listed in Table 3.

**Figure 3:**
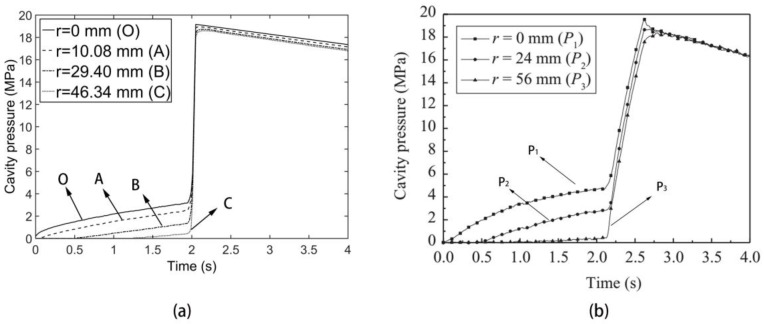
Pressure response for the conventional injection molding technology (CIMT) case. (**a**) Simulation result; and (**b**) experimental monitoring result [7].

**Figure 4:**
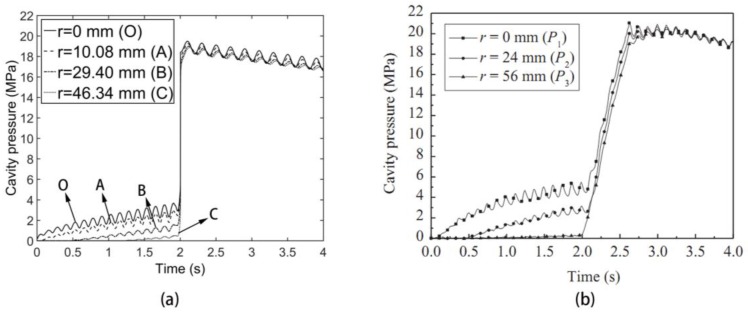
Pressure response for the dynamic injection molding technology (DIMT) case. (**a**) Simulation result; and (**b**) experimental monitoring result [7].

By finite computation, we can obtain the pressure response of these four monitors both for the CIMT case and DIMT case. From Figure 3 and Figure 4, it is clarified that, compared with the experimental observation, our pressure results are efficient, although there is a certain difference between the computation and experiment. The value of the simulation results is slightly lower than the experimental value. This difference is mainly because the material property and processing parameters of computation cannot be exactly the same as the actual experiment. However, this indicates that our computation method can be used as a simulation tool for investigating the mechanical evolution and the product quality under DIMT further.

**Figure 5:**
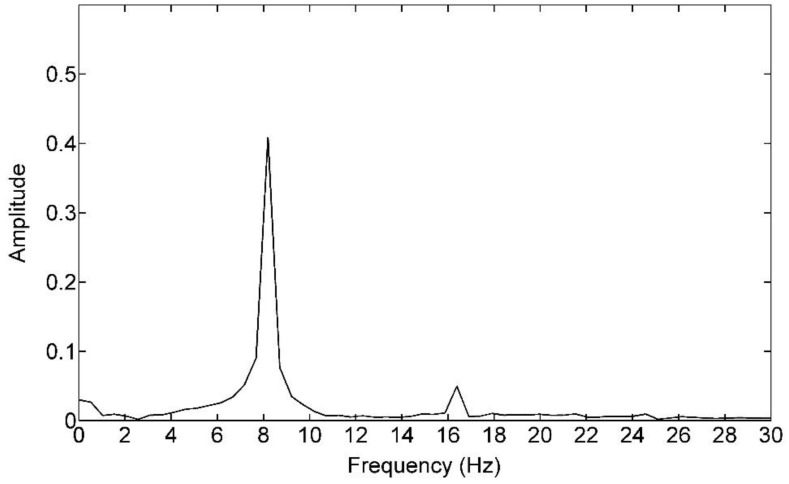
Spectrum of the pressure difference between the DIMT and CIMT.

There are several interesting phenomena in Figure 4. The first one is that the pressure response in the cavity has a fluctuation compared with the CIMT case. The second one is that this fluctuation amplitude gets larger and larger as time goes by for each monitor. The third one is that, although the observation of the pressure response rule for different monitors is the same, the further the monitor location is away from the gate, the smaller the fluctuation amplitude is. The fourth one is that, if we observe the fluctuation frequency, we find that this frequency seems like the one of the dynamic flow rate. If we make a fast Fourier transform (FFT) operation to this fluctuation, we can obtain the frequency domain as Figure 5, and the result shows that this fluctuation frequency is definitely the same as that of the flow rate. These observations exist in the filling phase. In the packing phase, the mean difference between these monitors is the fluctuation amplitude.

Recalling the finite element equation for computing the mechanical field under DIMT Equation (23), we can find the explanation of all of these phenomena. Actually, the entire solution of the equations not only includes the solution of the CIMT part, but also superposes the solution of the “vibration term”. For the solution of the CIMT part, it gives people the main trend of the pressure response curve, whereas the solution of the “vibration term” makes a certain pressure revision on the basis of the CIMT solution. For example, due to the periodic behavior of the flow rate in the dynamic filling phase, the higher flow rate of the DIMT case than the constant one of the CIMT case induces a pressure ascent, while the lower flow rate induces a pressure descent. This is the main reason that the pressure of the DIMT case fluctuates around the pressure of the CIMT case. For the difference of the fluctuation amplitude between these monitors, it is because the pressure value depends on the flow length between the node and the flow front. The larger the flow length is, the larger the fluctuation amplitude presents.

## 4. Pressure Response Analysis at the Gate

Pressure fluctuation exists in the cavity, while it is the most obvious at the gate. Therefore, we can focus on the injecting pressure to investigate the influence of DIMT parameters on the evolution of the pressure field.

### 4.1. Influence of the Filling Amplitude on the Transient Injecting Pressure

**Figure 6:**
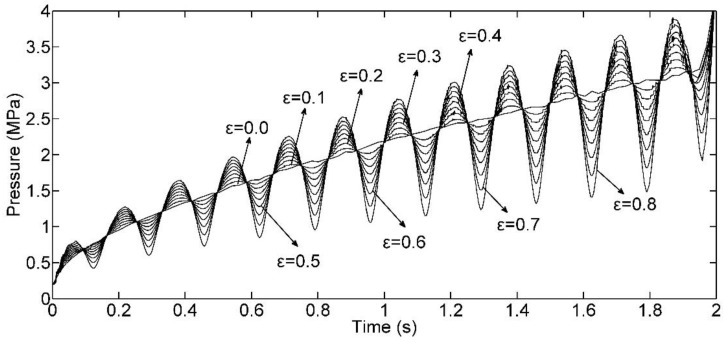
Injecting pressure curves with different *ε*.

**Figure 7:**
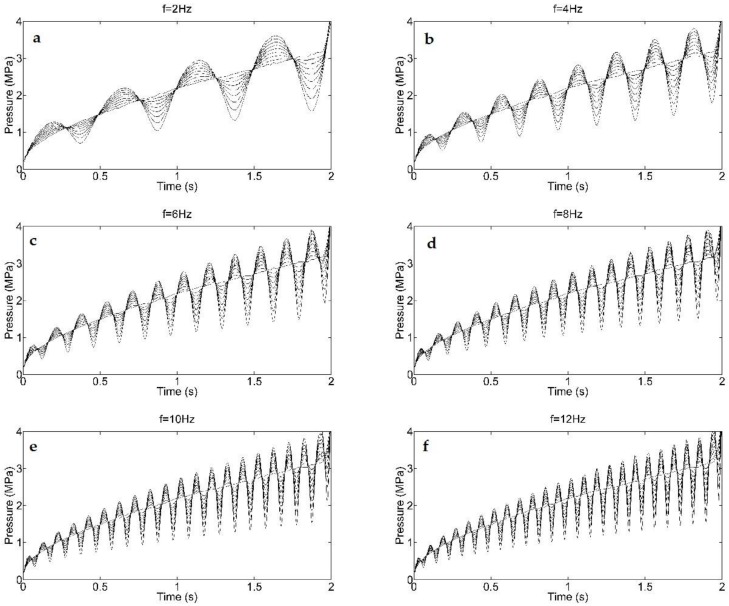
Injection pressure curves for different frequencies with the same ε as Figure 6. (**a**) *f* = 2 Hz; (**b**) *f* = 4 Hz; (**c**) *f* = 6 Hz; (**d**) *f* = 8 Hz; (**e**) *f* = 10 Hz; (**f**) *f* = 12 Hz.

In this subsection, we make *f_fill_* equal to 6 Hz, *φ_fill_* equals 0, then analyze that the transient injecting pressure response of nine different values for *ε* from 0 to 0.8 at 0.1 intervals. The injecting pressure curves are depicted as Figure 6. This shows that, as *ε* increases from the conventional case, the volatility level increases. This observation also exists for other cases of *f_fill_* as shown in Figure 7. This is because the pressure solution of DIMT is the revision of CIMT case from Equation (23), which was explained in Section 3. However, it also implies from the figure that if *ε* gets closer to 0, the “dynamic effect” will become inefficient, since the dynamic flow rate yields into the conventional case, and parameters *f_fill_* and *φ_fill_* become useless process parameters simultaneously.

### 4.2. Influence of the Filling Frequency on the Transient Injecting Pressure

**Figure 8:**
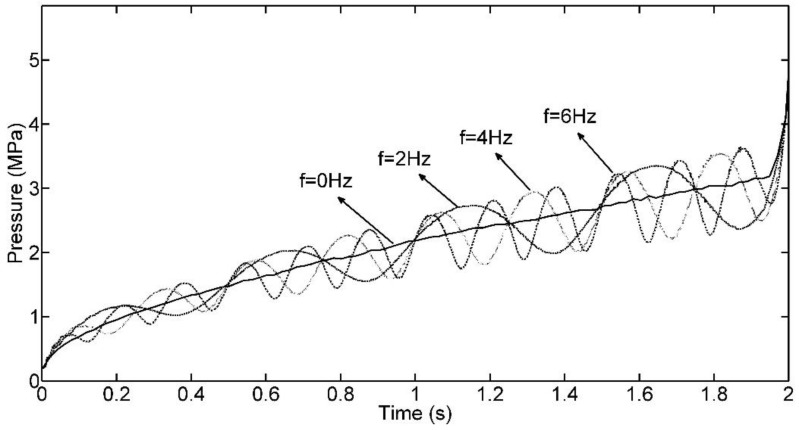
Injection pressure curves with different *f_fill_*.

In this subsection, we choose *ε* = 0.5, *φ_fill_* = 0, *f_fill_* belongs to [0, 12] Hz of 2 Hz interval, then, we simulate the filling process for each case of seven different values of *f_fill_*. The final transient pressure curves of the front four cases are collected in Figure 8. According to the analysis in Section 3, we know that *f_fill_* actually affects the frequency of the dynamic injecting pressure by using FFT technology. In Figure 8, it is notable that for the given *ε*, the entire volatility level of injecting pressure for each case is almost the same, even though *f_fill_* in each case is different. However, different *f_fill_* can cause different injecting pressures at the end of the filling phase. This is not a good expectation in some cases. For instance, if ε is close to 1 and *f_fill_* takes a small value, it will cause the short shot issue due to low injecting pressure when the flow rate descends below the mean value of the CIMT case at the late period of filling.

### 4.3. Influence of the DIMT Parameters on the Mean Injecting Pressure

From the previous observations, we know that pressure of DIMT fluctuates around the pressure curve of CIMT, sometimes higher and sometimes lower. It is not valuable to compare the pressure between DIMT and CIMT at any given time, but valuable to study the influence of dynamic parameters on the mean pressure during filling phase. The mean pressure during the filling phase represents the fluidity of the melt and the total input work, which can be defined as:

(32) $$\bar{p}=\frac{\int_{0}^{t_{\mathrm{fill}}}p\left(t\prime \right)dt\prime }{t_{\mathrm{fill}}}$$

**Figure 9:**
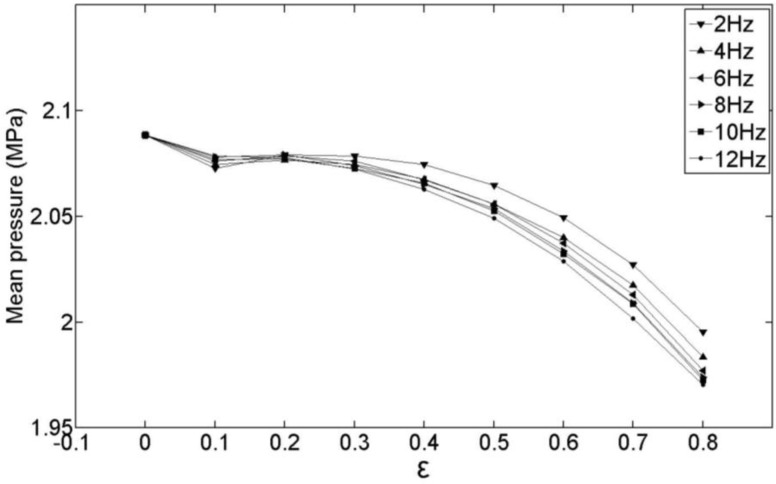
Mean filling pressure curves with different frequencies and amplitude factors.

By using Equation (32), we have computed the mean pressure of the different filling frequencies, and obtained the relationship between the mean pressure and *ε*, which is shown in Figure 9. Obviously, the mean pressure of the DIMT case is lower than that of the CIMT case from the figure. Moreover, as the amplitude increases, the mean pressure decreases for each one of the investigating frequency simulations. This indicates that the melt flows much easier under DIMT than CIMT, and more energy is saved for manufacturing the product. This is good news for producers since the energy saved is much more considerable for continuously manufacturing products. However, this effect is not obvious for ε belongs to [0, 0.3] since small *ε* induces an unimpressive fluctuation, as mentioned in Section 3.

**Figure 10:**
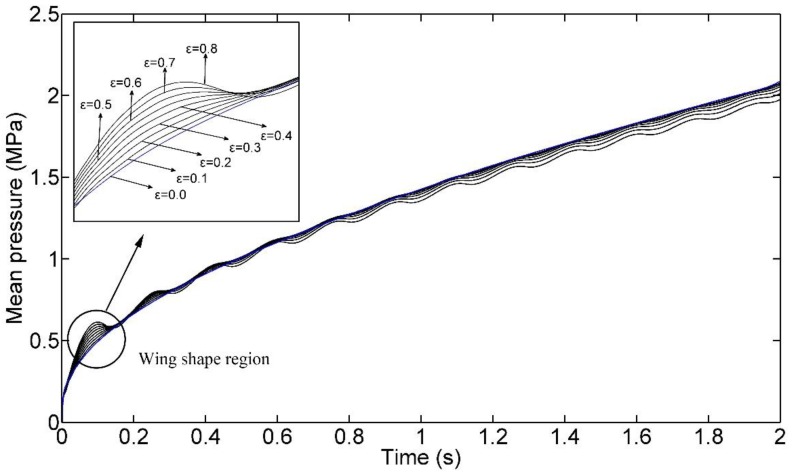
Time domain curves of the mean filling pressure.

**Figure 11:**
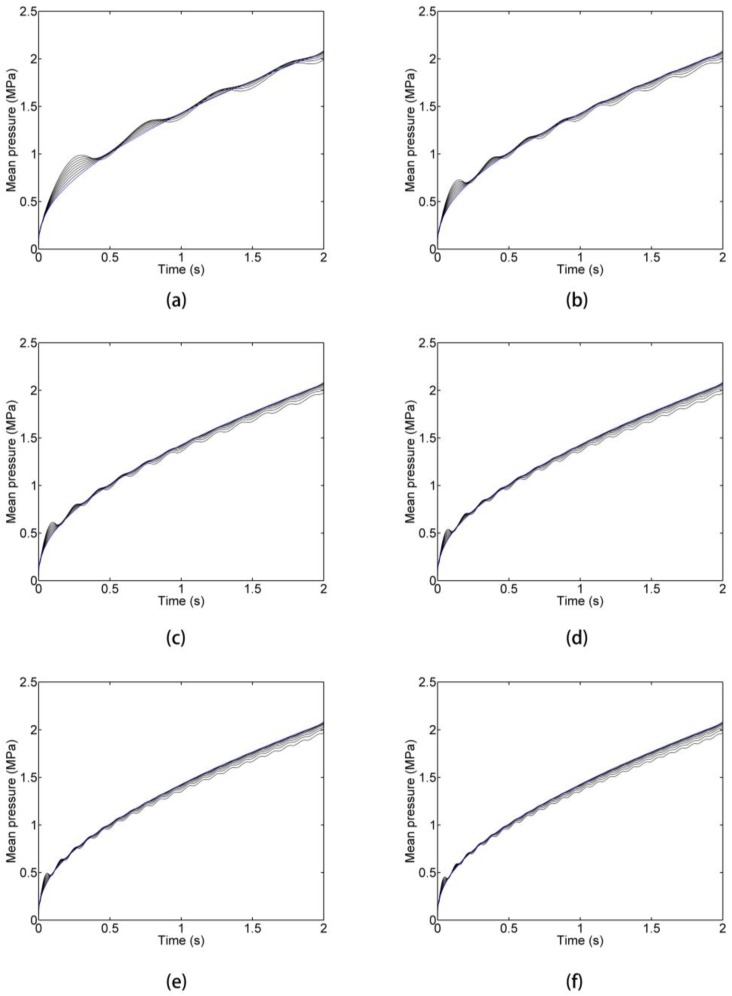
Time domain curves of the mean filling pressure with the same *ε* as figure 10. (**a**) *f* = 2 Hz; (**b**) *f* = 4 Hz; (**c**) *f* = 6 Hz; (**d**) *f* = 8 Hz; (**e**) *f* = 10 Hz; (**f**) *f* = 12 Hz.

If we change the upper limit of integral *t_fill_* into *t*, which belongs to [0, *t_fill_*] in Equation (32), we can observe the evolution of the mean pressure. For instance, Figure 10 presents the mean pressure curves for *f_fill_* = 6 Hz. In this figure, it reveals that the conclusion in Figure 9 of the above study cannot always hold during the entire filling process, but holds after a certain time in the filling process. At the beginning of the filling phase, there is a wing-shaped region which is enclosed by the mean pressure curve of DIMT and that of CIMT. The area of this region increases as *ε* increases. Moreover, for a given *ε*, the mean pressure curve presents a fluctuation. These phenomena also exist in other frequency cases, as shown in Figure 11.

(33) $$\bar{p}=\frac{\int_{0}^{t}p\left(t\prime \right)dt\prime }{t}$$

At the beginning of the filling phase, it shows that the flow rate is increasing and is larger than the conventional flow rate from Equation (28) for this study. Therefore, the greater *ε* can cause a greater difference between the pressure curves of DIMT and CIMT, which makes a wing-shaped region. As time goes on, the effect of DIMT emerges that reduces the flow resistance, and the mean pressure descends below that of CIMT.

In order to explain the mean pressure fluctuation observation, we need to analyze the mathematical monotony of the mean pressure curve, which is deduced from Equation (33) as:

(34) $$\frac{d\bar{p}\left(t\right)}{dt}=\frac{t\cdot p\left(t\right)-\int_{0}^{t}p\left(t\prime \right)dt\prime }{t^{2}}=\frac{F\left(t\right)}{t^{2}}$$

where $F\left(t\right)=t\cdot p\left(t\right)-\int_{0}^{t}p\left(t\prime \right)dt\prime$ determines the monotony of the mean pressure curve. By using the mean value theory of integral, which states:

(35) $$\begin{array}{cc} F\left(t\right) & =t\cdot p\left(t\right)-\int_{0}^{t}p\left(t\prime \right)dt\prime \\ & =t\cdot p\left(t\right)-t\cdot p\left(\tau \right)\\ & =t\cdot \left[p\left(t\right)-p\left(\tau \right)\right] \end{array}$$

where *τ* belongs to [0, t), and *τ* = 0 only if *t* = 0 for this problem. Then, Equation (34) is simplified as:

(36) $$\frac{d\bar{p}\left(t\right)}{dt}=\frac{t\cdot \left[p\left(t\right)-p\left(\tau \right)\right]}{t^{2}}$$

Equation (35) associates the monotony of the mean pressure with the injecting pressure value. For CIMT, it is constant practice that *p*(*τ*) < *p*(*t*) since the injecting pressure is monotone increasing for filling the cavity. However, for DIMT, it does not hold anymore. Recalling the observation of the injecting pressure of DIMT, *p*(*τ*) may be smaller than *p*(*t*), and *p*(*τ*) may be greater than *p*(*t*), as well, due to the pressure fluctuation of DIMT. Therefore, the monotony of the mean pressure for DIMT is variable, which induces the fluctuation phenomenon for all cases in Figure 11.

It can be clarified from another point of view that if computing the gradient of *F*(*t*) with respect to *t*, we can obtain:

(37) $$\begin{array}{cc} \frac{\mathrm{dF}\left(t\right)}{dt} & =\frac{d\left[t\cdot p\left(t\right)-\int_{0}^{t}p\left(t\prime \right)dt\prime \right]}{dt}\\ & =t\frac{\mathrm{dp}\left(t\right)}{dt} \end{array}$$

There is no doubt that *F*(0) = 0. Then, Equation (34) can be rewritten as:

(38) $$\begin{array}{cc} \frac{d\bar{p}\left(t\right)}{dt} & =\frac{t\cdot p\left(t\right)-\int_{0}^{t}p\left(t\prime \right)dt\prime }{t^{2}}\\ & =\frac{\int_{0}^{t}t\prime \frac{\mathrm{dp}\left(t\prime \right)}{dt\prime }dt\prime }{t^{2}} \end{array}$$

Using the mean value theory of integral again, we can deduce a much simpler formula:

(39) $$\frac{d\bar{p}\left(t\right)}{dt}=\frac{\tau \cdot \dot{p}\left(\tau \right)}{t}$$

Equation (39) associates the monotony of the mean pressure with the monotony of the injecting pressure. It can illustrate the phenomena in Figure 11 more directly with the help of the observations on the injecting pressure response.

## 5. Product Warpage Optimization Design of Different Structure Types

### 5.1. Optimization Model

For thin-walled products, warpage is one of the most important molding defects. In the following sections, the purpose is to minimize the warpage of thin-walled products and argue the actual effect of DIMT compared with CIMT. The warpage optimization model is proposed as:

(40) $$\{\begin{array}{l} Find:\;x=\left[x^{1},x^{2},x^{3},\ldots ,x^{N_{\mathrm{var}}}\right]\\ Min:\;y\left(x\right)=\max\sqrt{d_{x}^{i}{\left(x\right)}^{2}+d_{y}^{i}{\left(x\right)}^{2}+d_{z}^{i}{\left(x\right)}^{2}}\;i=1,2,\ldots ,N_{node}\\ S.T.:\;x_{lb}^{i}\leq x^{i}\leq x_{ub}^{i},\;x^{i}\in x,\;i=1,2,\ldots ,N_{\mathrm{var}} \end{array}$$

where $d_{x}^{i}$(***x***), $d_{y}^{i}$(***x***), and $d_{z}^{i}$(***x***) represent the deformation in the *x*, *y*, and *z* direction of node *i*. *N_node_* and *N*_var_ represent the node number and the variable number, respectively.

In order to compare the improvement level of warpage, the process parameter optimizations for CIMT and DIMT are both executed. For CIMT, melt temperature *x*^1^ (*T_melt_*), mold temperature *x*^2^ (*T_mold_*), filling time *x*^3^ (*t_fill_*), packing time *x*^4^ (*t_pack_*), initial packing fraction *x*^5^ (*P_start_*), and trend coefficient *x*^6^ (*δ*) are selected as the optimization parameters. For DIMT, except the previous six parameters, filling amplitude *x*^7^ (*ε*), filling frequency *x*^8^ (*f_fill_*), filling phase *x*^9^ (*φ_fill_*), packing amplitude *x*^10^ (*θ*), packing frequency *x*^11^ (*f_pack_*), and packing phase *x*^12^ (*φ_pack_*) are chosen, additionally.

The optimization objective in Equation (40) is a “black” function with respect to **x**. In this paper, a Kriging surrogate model [26,27] is taken into consideration to evaluate the objective approximately, since it can give us more prediction information, and a maximum experiment improvement (MEI) function-based sequential optimization method [28] is employed to solve Equation (40). The detailed contents of the optimization method and procedure can be found in Appendix A. Then, the equal optimization issue changes to be:

(41) $$\{\begin{array}{l} Find:\;x=\left[x^{1},x^{2},x^{3},\ldots ,x^{N_{\mathrm{var}}}\right]\\ Max:\;EI\left(x\right)\\ S.T.:\;x_{lb}^{i}\leq x^{i}\leq x_{ub}^{i},\;x^{i}\in x,\;i=1,2,\ldots ,N_{\mathrm{var}} \end{array}$$

with the convergence criterion:

(42) $$\frac{EI^{I}}{y_{\max}^{I}-y_{\min}^{I}}\leq {ο}_{c}$$

where $y_{max}^{I}$ and $y_{m\mathrm{in}}^{I}$ are the maximum and minimum response values in the sample set of the *I*_th_ searching for Equation (41), respectively. Herein, *o_c_* is 0.001 in this paper.

### 5.2. Structure and Parameters

In this section, three types of warpage optimization issues are studied to argue the improvement capability of DIMT compared with CIMT.

#### (a) Small Size Product with No Holes

**Figure 12:**
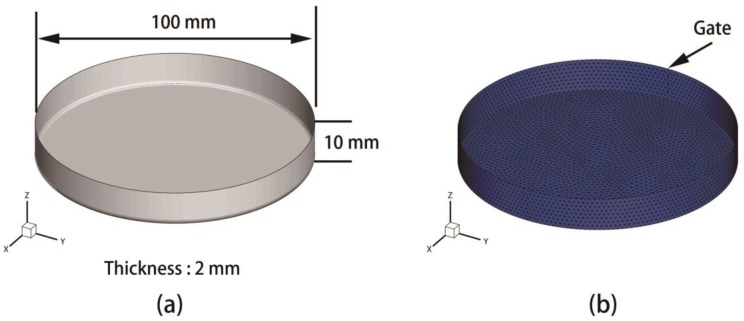
Polycarbonate light cover. (**a**) Geometry; (**b**) Finite element model.

The first product model is a polycarbonate (PC, the type is X-1) light cover with no holes in it. The geometry and the finite element model of the product are sketched as shown in Figure 12. The basic properties of the molding material are listed in in detail Table 4. The value ranges of 12 process parameters are tabulated as Table 5. For the optimization of CIMT, the design variables involved are the first six variables in Table 5.

#### (b) Small Size Product with Multiple Holes

**Figure 13:**
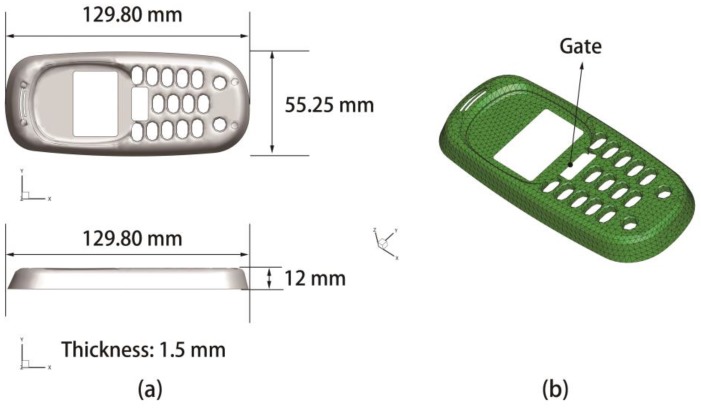
Geometry and finite element model of the PC/ABS phone cover. (**a**) Geometry; (**b**) Finite element model.

The second product model is a phone cover used in our previous work [20], which is made by the blend of PC and acrylonitrile butadiene styrene (ABS) copolymers. Herein, it is only combined as a comparison with other issues. In this case, there are multiple holes in the product compared with issue (a). Figure 13 is the geometry and the finite element model of the product. The basic properties of the molding material are listed in Table 6. The value ranges of 12 process parameters are tabulated as Table 7, in which the design variables involved in CIMT optimization issue are the first six variables.

#### (c) Large, Narrow Product

**Figure 14:**
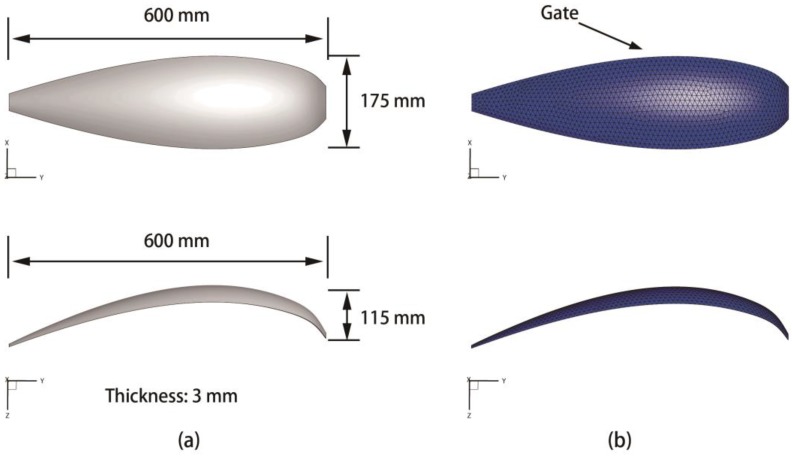
Geometry and finite element model of the car light cover. (**a**) Geometry; (**b**) Finite element model.

The third product is a large, narrow PC light cover. The geometrical structure information and finite element model of this PC cover is sketched in Figure 14. The basic properties information of the molding material is listed in Table 8. The value ranges of 12 process parameters are tabulated as Table 9. For the CIMT case, the design variables take the first six variables in Table 9.

### 5.3. Results and Discussions

**Figure 15:**
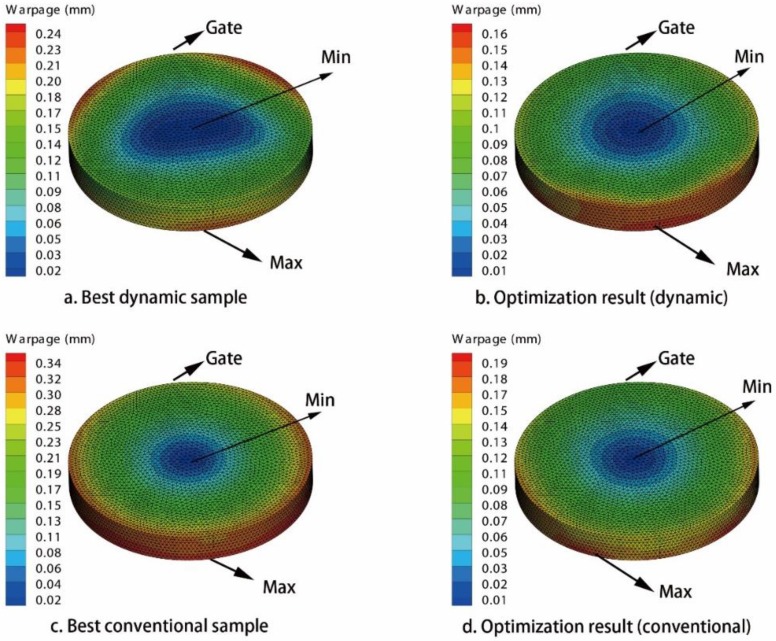
Warpage contours of the PC light cover. (**a**) Best dynamic sample; (**b**) optimization result of the dynamic issue; (**c**) best conventional sample; (**d**) optimization result of the conventional issue.

**Figure 16:**
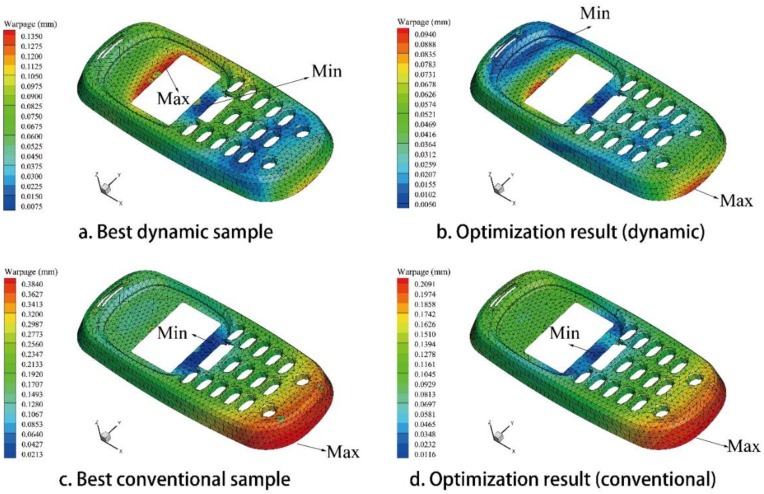
Warpage contours of the PC/ABS cellular phone cover model. (**a**) Best dynamic sample; (**b**) optimization result of the dynamic issue; (**c**) best conventional sample; and (**d**) optimization result of the conventional issue.

**Figure 17:**
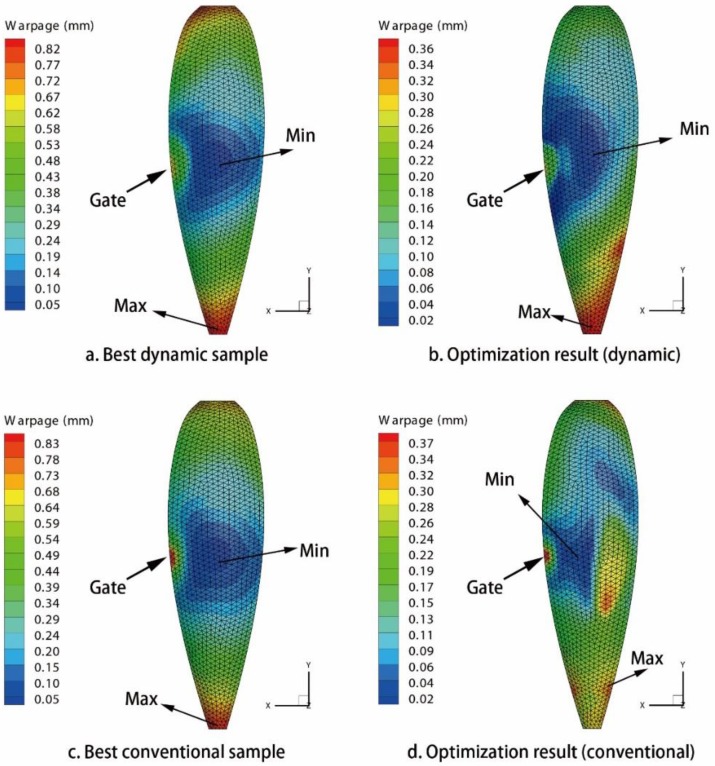
Warpage contours of the PC car light cover model. (**a**) Best conventional sample; (**b**) optimization result of the conventional issue; (**c**) the best dynamic initial sample; and (**d**) the optimization result of the dynamic issue.

By using MEI-based optimization method in Appendix A, all of the issues of warpage optimization are solved. The optimization results of three CIMT cases are listed in Table 10, Table 12, and Table 14, respectively. The optimization results of three DIMT cases are listed in Table 11, Table 13, and Table 15, respectively. For issue (a), the warpage of the PC light cover under DIMT (0.168 mm) is smaller than that of the CIMT case (0.197 mm), which is reduced by 14.72%. For issue (b), the warpage of the PC/ABS phone cover under DIMT (0.094 mm) is smaller than that of the CIMT case (0.221 mm), which is reduced by 57.47%. However, for issue (c), the warpage of the PC car light cover under DIMT (0.375 mm) is almost the same as that of the CIMT case (0.388 mm). This shows that DIMT do not improve the molding quality better than CIMT for this large narrow product. The warpage contour figures of all of the results are organized in Figure 15, Figure 16 and Figure 17, respectively.

For these three issues, the results show that DIMT can greatly reduce the warpage of the products, even though the structure and molding material are different. For issues (a) and (c), the dynamic parameters are all “efficient”. However, the *f_pack_* and *φ_pack_* are both “inefficient” in issue (b) since the packing amplitude is 0, and the molding process yields into the CIMT case during packing phase. This is to say that, in issue (b), only dynamic filling parameters worked. The discussion of this optimization result is not the main work here, and has been stated in our previous work [20].

From the warpage contours, it shows that, in issues (a) and (b), the distribution area of the smaller warpage region under DIMT are larger than that of the CIMT cases, and the distribution area of the larger warpage region under DIMT is smaller than that of the CIMT cases. Therefore, DIMT can not only reduce warpage much more than CIMT, but also improve the entire molding quality for the small size products. Unfortunately, it does not happen for issue (c), which makes the molding quality much worse than CIMT. We should recall the pressure response observation of the cavity in Section 3. From the analysis in Section 3, we should realize that the pressure volatility relies on the flow length. If the geometry size is large, at the location far away from the gate, the pressure will not be affected by the vibration. This causes a great difference between the region around the gate and the region away from the gate. It may not have a good mechanical history for improving the molding quality. Therefore, it fails.

Actually, the in-cavity residual stress is the final result of the thermal and mechanical evolution before ejection. The uneven distribution of the in-cavity residual stress is the only reason for the warpage after ejection. DIMT takes an external force into the flow history. For small size product, it can disturb the flow behavior and may eliminate the magnitude and distribution difference of the in-cavity residual stress, which results in a lower warpage level compared with CIMT. Whereas, for large-sized, narrow products, this contribution is limited around the gate, and may not be good for improving the molding quality, even making it worse. Therefore, using DIMT should be seriously considered to produce the polymer product.

## 6. Conclusions

In this paper, the numerical DIMT is proposed by using the finite element method. More detailed work has been performed for observing and analyzing the mechanical evolution behavior of DIMT. The effect of dynamic parameters on the transient pressure of the cavity and the mean pressure of the gate are analyzed for realizing the mechanical behavior of DIMT. It is also fundamental for explaining the mechanism in the following warpage optimization work.

Three kinds of optimization issues with different molding materials are constructed in this paper. By using the EGO sequential optimization method, warpage values of all of the products are efficiently reduced. The further analysis and discussions reveal that DIMT is not available for all types of structures, especially for the large-scale, narrow structures. The main reason for this is that the thermal and mechanical evolution history can determine the in-cavity residual stress in the product before being ejected out of the mold. For small-sized products, dynamic process parameters make the key contribution to disturb the in-cavity residual stress distribution, which can finally increase the region of lower warpage and reduce the warpage difference in the product. Whereas, for large and narrow products, the dynamic contribution concentrates around the gate. It makes a great mechanical difference for this kind of structure, and causes a significant difference in warpage in the product. Therefore, as a recommendation, for this type of product, DIMT should be seriously considered. Overall, the content of this study focuses on revealing the mechanical mechanism of DIMT, and may be a guide for the actual manufacturing with DIMT.

## Figures and Tables

**Table 1:** Viscosity models for describing the non-Newtonian behavior of plastic melt [21,22,23].

Model	Contributor
$\eta =K\cdot {\dot{\gamma }}^{n-1}$	De Waele (1923) and Ostward (1925)
$\eta =\frac{\eta_{0}}{1+\sigma_{sh}/G_{i}}$	Ferry (1942)
$\eta =\frac{\eta_{0}}{1+{\left(\tau \prime \dot{\gamma }\right)}^{m}}$	Cross (1965,1968)
$\eta =\frac{\eta_{0}}{1+{\left(\frac{\eta_{0}\dot{\gamma }}{\tau^{*}}\right)}^{1-n}}$	Willianms, Landel and Ferry (1955)
$\eta =\eta_{0}{\left[1+{\left(\tau \prime \dot{\gamma }\right)}^{a}\right]}^{\left(n-1\right)/a}$	Yasuda, Armstrong and Cohen (1981)

**Table 2:** Basic properties of LDPE.

Property	Solid density g∙cm^−3^	Melt density g∙cm^−3^	Thermal conductivity W∙m^−1^∙°C^−1^	Poison ratio	Transition temperature °C
Value	0.920	0.759	0.31	0.41	90

**Table 3:** Dynamic injection molding parameters used in this study.

Parameter	Symbol	Value
Packing pressure fraction (%)	*P_start_*	330
Trend coefficient	*δ*	16
Filling amplitude	*ε*	0.5
Filling frequency (Hz)	*f_fill_*	8
Initial filling phase	*φ_fill_*	0
Packing amplitude	*θ*	10
Packing frequency (Hz)	*f_pack_*	5
Initial packing phase	*φ_pack_*	3π/2

**Table 4:** Basic properties of PC X-1.

Melt density g∙cm^−3^	Solid density g∙cm^−3^	Specific heat J∙kg^−1^∙K^−1^	Elastic modulus MPa	Poison ratio	Coefficient of thermal expansion °C^−1^
1.062	1.192	1964	2280	0.417	7.3 × 10^−5^

**Table 5:** The boundary values of the design parameters.

Parameter	*T_melt_* °C	*T_mold_* °C	*t_fill_* s	*t_pack_* s	*P_start_* %	*δ*	*ε*	*f_fill_* Hz	*φ_fill_*	*θ*	*f_pack_* Hz	*φ_pack_*
Upper	293	120	1.5	4	90	10	1	50	2π	30	50	2π
Lower	260	70	0.5	1	60	0	0	0	0	0	0	0

**Table 6:** Basic properties of PC/ABS.

Melt density g∙cm^−3^	Solid density g∙cm^−3^	Specific heat J∙kg^−1^∙K^−1^	Elastic modulus MPa	Poison ratio	Coefficient of thermal expansion °C^−1^
1.1092	1.2038	1159	2780	0.4	6.7 × 10^−5^

**Table 7:** The boundary values of the design parameters.

Parameter	*T_melt_* °C	*T_mold_* °C	*t_fill_* s	*t_pack_* s	*P_start_* %	*δ*	*ε*	*f_fill_* Hz	*φ_fill_*	*θ*	*f_pack_* Hz	*φ_pack_*
Upper	300	92	1	5	90	10	1	20	2π	30	50	2π
Lower	230	50	0.2	0.5	60	0	0	0	0	0	0	0

**Table 8:** Basic properties of PC Lexan.

Melt density g∙cm^−3^	Solid density g∙cm^−3^	Specific heat J∙kg^−1^∙K^−1^	Elastic modulus MPa	Poison ratio	Coefficient of thermal expansion °C^−1^
1.046	1.193	1881	2280	0.417	7.3 × 10^−5^

**Table 9:** The boundary values of the design parameters.

Parameter	*T_melt_* °C	*T_mold_* °C	*t_fill_* s	*t_pack_* s	*P_start_* %	*δ*	*ε*	*f_fill_* Hz	*φ_fill_*	*θ*	*f_pack_* Hz	*φ_pack_*
Upper	320	120	5	8	90	10	1	50	2π	30	50	2π
Lower	280	80	1.5	3	60	0	0	0	0	0	0	0

**Table 10:** Conventional parameters and warpage results of the PC light cover.

Parameter	*T_melt_* °C	*T_mold_* °C	*t_fill_* s	*t_pack_* s	*P_start_* %	*δ*	Warpage mm
Initial	268.23	83.85	1.33	1.67	86.40	1.67	0.362
Optimized	260	89.05	1.50	2.52	90	0.6	0.197

**Table 11:** Dynamic parameters and warpage results of the PC light cover.

Parameter	*T_melt_* °C	*T_mold_* °C	*t_fill_* s	*t_pack_* s	*P*_start_ %	*δ*	*ε*	*f_fill_* Hz	*φ_fill_*	*θ*	*f_pack_* Hz	*φ_pack_*	Warpage mm
Initial	262.81	75.62	0.64	3.56	84.76	2.22	0.25	13.85	1.91	20.65	33.04	3.98	0.257
Optimized	265.79	97.50	1.20	3.58	88.79	7.44	0.92	10.42	4.55	12.19	28.84	2.12	0.168

**Table 12:** Conventional parameters and warpage results of the PC/ABS phone cover.

Parameter	*T_melt_* °C	*T_mold_* °C	*t_fill_* s	*t_pack_* s	*P_start_* %	*δ*	Warpage mm
Initial	268.65	73.42	0.67	4.89	72.11	5.46	0.405
Optimized	250.86	50	0.40	2.12	82.59	10	0.221

**Table 13:** Dynamic parameters and warpage results of the PC/ABS phone cover.

Parameter	*T_melt_* °C	*T_mold_* °C	*t_fill_* s	*t_pack_* s	*P_start_* %	*δ*	*ε*	*f_fill_* Hz	*φ_fill_*	*θ*	*f*_pack_ Hz	*φ*_pack_	Warpage mm
Initial	262.43	74.71	0.96	1.95	84.41	7.55	0.64	0.59	0.13	3.63	24.41	5.73	0.131
Optimized	277.82	77.06	0.72	3.31	82.86	10.00	0.32	11.39	1.67	0.00	48.74	1.74	0.094

**Table 14:** Conventional parameters and warpage results of the PC car light cover.

Parameter	*T_melt_* °C	*T_mold_* °C	*t_fill_* s	*t_pack_* s	*P_start_* %	*δ*	Warpage mm
Initial	289.98	91.08	4.39	4.11	86.40	1.67	0.879
Optimized	291.84	90.89	4.00	6.58	90	0.33	0.388

**Table 15:** Dynamic parameters and warpage results of the PC car light cover.

Parameter	*T_melt_* °C	*T_mold_* °C	*t_fill_* s	*t_pack_* s	*P_start_* %	*δ*	*ε*	*f_fill_* Hz	*φ_fill_*	*θ*	*f_pack_* Hz	*φ_pack_*	Warpage mm
Initial	283.40	84.50	1.99	7.26	84.76	2.22	0.25	13.85	1.91	20.65	33.04	3.98	0.865
Optimized	288.46	88.25	2.36	6.48	89.11	0.92	0.39	23.56	2.55	10.93	14.36	3.95	0.375

## Appendix A. Kriging Surrogate and EI-Based Sequential Optimization Method (Equation Section (Next))

An optimization issue of the black function *y*(***x***) is given by:

(A1) $$\{\begin{array}{l} Find:\;x=\left[x^{1},x^{2},x^{3},\ldots ,x^{m}\right]\\ Min:\;y\left(x\right)\\ \begin{array}{l} S.T.:\;x_{lb}^{i}\leq x^{i}\leq x_{ub}^{i},\;x^{i}\in x,\;i=1,2,\ldots ,m\\ \;g_{j}\left(x\right)\leq 0,\;j=1,2,\ldots ,N_{g}\\ \;h_{k}\left(x\right)=0,\;k=1,2,\ldots ,N_{h} \end{array} \end{array}$$

where *m* is the number of the variable, and *N_g_* and *N_h_* are the numbers of the constraints.

It needs two datasets for constructing the kriging model for Equation (A1), the sample set ***X*** of the design variables and the corresponding objective value set ***Y***. For the given variable sample set ***X*** and the corresponding objective set ***Y***, there are two parts, a polynomial part and a stochastic function, in the kriging model shown in Equation (A2):

(A2) $$y(x)=\sum_{k=1}^{l}\beta_{k}f_{k}(x)+z(x)$$

where *β_k_* and *f_k_*(***x***) are the *k*th regression coefficient and polynomial. *z*(***x***) is the Gaussian stochastic function which follows norm [0, σ^2^]. For ***x****_i_* and ***x****_j_*, *z*(***x****_i_*) and *z*(***x****_j_*) are correlated with the spatial weighted distance between ***x****_i_* and ***x****_j_*. The covariance is expressed as:

(A3) $$Cov\left[z(x_{i}),z(x_{j})\right]=\sigma^{2}\cdot R_{ij}(\lambda ,x_{i},x_{j})$$

where *R_ij_* is a correlation function. We usually employ Gaussian form as shown in Equation (A4):

(A4) $$R_{ij}(\lambda ,x_{i},x_{j})=\prod_{n=1}^{m}\exp\left[-\lambda^{n}{\left(x_{i}^{n}-x_{j}^{n}\right)}^{2}\right]$$

where *λ_n_* is the *n*th correlated coefficient for the *n*th variables.

The ordinary kriging (OK) model is commonly used in most studies, which *f_k_*(***x***) in Equation (A2) only employs the zero-order. The prediction and variance of the OK model at any point **x*** yields:

(A5) $$\bar{y}(x*)=u+r^{T}(x*)\cdot R^{-1}\left(Y-u\cdot l\right)$$

(A6) $${\tilde{s}}^{2}(x*)={\tilde{\sigma }}^{2}\left[1+\frac{{\left(1-l^{T}R^{-1}r(x*)\right)}^{2}}{l^{T}R^{-1}l}-r^{T}(x*)R^{-1}r(x*)\right]$$

where *u* = (***l***^T^ ***R***^−1^***l***)^−1^(***l***^T^ ***R***^−1^***Y***). ***Y*** is the objective vector. *r*_i_ = *R*(*λ*, ***x****, ***x****_i_*). R is correlation function matrix. The element of ***l*** equals one. ${\tilde{\sigma }}^{2}$ is the maximum likelihood of $\sigma^{2}$.

According to the properties of kriging model, we can define some optimal searching functions to exploiting the solution of problem Equation (40). The most efficient searching function is the expected improvement (EI) function, which is the expectation of the *I*(***x***) = *y*_min_ − *y*(***x***) in the positive region of *I*(***x***). The detail formula of EI function is defined as:

(A7) $$E\left[I(x)\right]=\tilde{s}(x)\phi \left[\frac{y_{\min}-\bar{y}(x)}{\tilde{s}(x)}\right]+\left[y_{\min}-\bar{y}(x)\right]\Phi \left[\frac{y_{\min}-\bar{y}(x)}{\tilde{s}(x)}\right]$$

where *y*_min_ is the minimum value in the current sample set ***Y***.

In mathematics, *E*[*I*(***x***)] is an expectation surface to exploit a smaller design than the minimum in the current sample response set, or the most uncertain space design point from the expression Equation (A7). Therefore, Equation (A7) has both the local and global searching ability, and the maximum of *E*[*I*(***x***)] (MEI) contains the strongest potential to find the optimal solution, or points out the lack of information of the current surrogate model. This means that there exists little space for improvement to find a better design point than the current one, or to support a valuable spatial point to revise the current surrogate model. The MEI-based optimization method is usually called the efficient global optimization (EGO). Herein, we employ the MEI-based optimization method to solve the warpage optimization problem. Then the optimization problem changes into:

(A8) $$\{\begin{array}{l} Find:\;x=\left[x^{1},x^{2},x^{3},\ldots ,x^{m}\right]\\ Max:\;EI\left(x\right)\\ \begin{array}{l} S.T.:\;x_{lb}^{i}\leq x^{i}\leq x_{ub}^{i},\;x^{i}\in x,\;i=1,2,\ldots ,m\\ \;g_{j}\left(x\right)\leq 0,\;j=1,2,\ldots ,N_{g}\\ \;h_{k}\left(x\right)=0,\;k=1,2,\ldots ,N_{h} \end{array} \end{array}$$

The optimizing procedure is stated as:

(a) Select the proper initial sample design ***X*** by using the mathematical sampling method, like orthogonal Latin hypercube sampling, optimized Latin hypercube sampling. etc., and compute the corresponding objective set ***Y***;
(b) Construct the kriging surrogate model with ***X*** and ***Y***;
(c) Construct the EI function and the optimization problem Equation (A8), and deal with the constraint conditions;
(d) Find the optimal design ***x**** of problem Equation (A8) by using any mathematical optimization method, like GA, SQP, etc., and compute the corresponding objective value *y**;
(e) Justify whether the design [***x****, *y**] satisfies the convergence condition Equation (A9). If not, add [***x****, *y**] into [***X***, ***Y***], then recall steps (b)–(e), until the convergence condition is satisfied; If yes, optimization can finish:
  (A9) $$\frac{EI^{i}}{y_{\max}-y_{\min}}\leq {ο}_{c}$$

From the procedure, it is said that solving Equation (A8) is not executed once, but executed again and again. Each solving needs to reconstruct the kriging model and EI function. Therefore, it reveals the nature that the MEI-based optimization method is a method of sequential modeling, indirectly optimizing the original problem.
